# Folate Receptor-Targeted and GSH-Responsive Carboxymethyl Chitosan Nanoparticles Containing Covalently Entrapped 6-Mercaptopurine for Enhanced Intracellular Drug Delivery in Leukemia

**DOI:** 10.3390/md16110439

**Published:** 2018-11-08

**Authors:** Xuan Wei, Jianhong Liao, Zahra Davoudi, Hua Zheng, Jingru Chen, Dan Li, Xiong Xiong, Yihua Yin, Xiuxiang Yu, Jinghui Xiong, Qun Wang

**Affiliations:** 1 Department of Pharmaceutical Engineering, School of Chemistry, Chemical Engineering and Life Science, Wuhan University of Technology, Wuhan 430070, China; qwqunwang@yahoo.com (X.W.); wqwangqun@hotmail.com (J.L.); uniteddealer@yahoo.com (J.C.); qwqunwang@gmail.com (D.L.); uniteddealer@gmail.com (X.X.); iamnanomacro@gmail.com (Y.Y.); 2 Department of Chemical and Biological Engineering, Iowa State University, Ames, IA 50011, USA; davoudi@iastate.edu; 3 Hubei Provincial Key Laboratory of Antiviral Drugs, Wuhan East Lake High-Tech Development Zone, Wuhan 430070, China; yuxiuxiang1970@163.com (X.Y.); xiongjinghui@gmail.com (J.X.)

**Keywords:** leukemia, chemotherapy, nanoparticles, drug release, GSH-sensitive, anticancer drug, folate receptor, chitosan, tumor, surface modification

## Abstract

For enhanced intracellular accumulation of 6-mercaptopurine (6-MP) in leukemia, a folate receptor-targeted and glutathione (GSH)-responsive polymeric prodrug nanoparticle was made. The nanoparticles were prepared by conjugating 6-MP to carboxymethyl chitosan via a GSH-sensitive carbonyl vinyl sulfide linkage, ultrasonic self-assembly and surface decoration with folate. The TEM graphs shows that the as-synthesized nanoparticles are spherical with a particle size of 170~220 nm. In vitro drug release of nanoparticles demonstrated acceptable stability in PBS containing 20 μM GSH at pH 7.4. However, the cumulative drug release rate of the samples containing 20 mM and 10 mM GSH medium reached 78.9% and 64.8%, respectively, in pH 5.0 at 20 h. This indicated that this nano-sized system is highly sensitive to GSH. The inhibition ratio of folate-modified nanoparticles compared to unmodified nanoparticles was higher in cancer cells (human promyelocytic leukemia cells, HL-60) while their cytotoxicity was lower in normal cells (mouse fibroblast cell lines, L929). Furthermore, in vitro cancer cell incubation studies confirmed that folate-modified nanoparticles therapeutics were significantly more effective than unmodified nanoparticles therapeutics. Our results suggest that folate receptor-targeting and GSH-stimulation can significantly elevate tumour intracellular drug release. Therefore, folate-modified nanoparticles containing chemoradiotherapy is a potential treatment for leukemia therapy.

## 1. Introduction

Over the past few years, chemotherapy has become the most accepted treatment of malignancy. Although antitumor drugs have the ability to inhibit the proliferation of cancer cells, the free spread of these small molecules to normal organs may result in serious systematic toxicity after long-term usage [1,2,3,4]. Different approaches have been employed to improve the therapeutic efficiency of anticancer drugs [5,6,7]. Among them, Glutathione (GSH)-sensitive polymeric nanoparticles for tumour-targeted delivery of anticancer drugs have attracted more and more attention in recent years due to several advantages, such as improving the stability and solubility of drugs, avoiding premature leaking in the blood stream and selective drug release enhancement in target tissues or cells, and increasing the circulation time in the physiological environment etc. [8,9].

GSH is a three-peptide containing sulfhydryl group, which exists in almost all mammalian tissues. Comparing the GSH concentration in tumour cells and normal cells, it has been shown that its concentration is much higher (at least four times) in tumour cells (2–10 mM) while its concentration is only in the micro molar range (2–20 μM) in blood plasma [10,11]. This discrepancy results in disulfide bond stability in the circulatory system and the possibility of more breakage in tumour cells. Due to this fact, most GSH-triggered drug delivery systems are obtained by introducing disulfide-bonded functionalized links into polymer carriers to control drug release in tumor cells [12,13], as well as exhibiting excellent anticancer properties. However, the carriers still have the following disadvantages: (1) Receptor-based active targeting to the tumour cell has not been considered; (2) Drug leakage and premature drug release hinder the system working at its maximum level; and (3) The comparison of the treated cells with normal cells in terms of cytotoxicity has been carried out in a very few studies [14,15].

The most important advantage of conjugating anticancer drugs to polymers via GSH-sensitive chemical bonds is that drug leakage or premature release can be efficiently avoided. Moreover, selective drug release can be achieved because of the difference in the GSH concentrations in tumour cells and normal cells. Considering the thiol group in the 6-mercaptopurine (6-MP) structure, we have previously constructed polymer prodrugs based on the disulfide bond or α, β-unsaturated carbonyl group [16,17]. The amphiphilic macromolecular prodrug conjugates (6-MP-CMCS) were prepared by grafting 6-MP onto carboxymethyl chitosan (CMCS) via disulfide or the α, β-unsaturated carbonyl group. Then, the self-assembly of 6-MP-CMCS into GSH-sensitive nanoparticles in an aqueous solution made the system suitable for leukemia treatment. However, the prepared prodrug conjugates have multiple drawbacks, including lack of drug targeting to the tumour cells and lack of central release in tumour cells.

Folate receptor (FR) is a high-affinity receptor that mediates the cellular uptake of folic acid and its derivative in the eukaryotic cells [18]. Compared with normal tissues, it has high expression in tumour tissues, which makes it a desired option for tumour targeted therapy [19]. Derivation of folate using one of its two carboxyl groups does not change its high affinity to FR [20,21]. Folate conjugation, therefore, provides a substrate for FR-targeted drug delivery. This method has been successfully applied to small molecules, macromolecules, and nanocarriers [22,23,24]. There are three types of folate receptors, of which FR-β has been found in myeloid leukemia. The over-expression of FR-β can be potentially used for targeted therapy of myeloid leukemia [25]. For example, smart human-serum-albumin—As_2_O_3_ nanodrug using this potential of FR-β has recently been reported, which could specifically recognize chronic myeloid leukemia cells and promote intracellular accumulation of As_2_O_3_. Therefore, a folate-targeted nanosystem focused on FR-β expression could enhance the targeting ability of leukemia therapy [26].

Chitosan is a natural polysaccharide consisting of N-acetyl glucosamine and glucosamine. It has excellent biological properties, including biodegradability, non-toxicity, low immunogenicity, and biocompatibility [27,28,29,30]. These features make chitosan a promising polymer vector for drug delivery systems [31]. However, because of its low solubility at physiological pH (above 6.0), its application is limited [32,33]. Carboxymethyl chitosan is a derivative formed by carboxymethylation of chitosan with chloroacetic acid, which retains some advantages of chitosan and also shows acceptable aqueous solubility in addition to improved flexibility [7,34,35]. Moreover, the final carboxymethyl groups can link to 6-MP via a connecting arm.

**Scheme 1:**
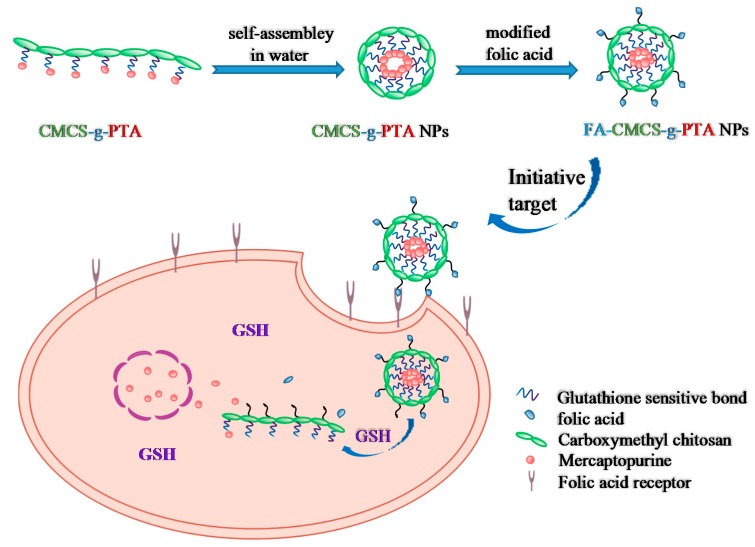
Schematic illustration of formation of FA-CMCS-g-PTA NPs and their intracellular uptake and GSH-sensitive release of 6-MP.

In this research, a novel GSH-responsive polymer prodrug nanoparticle (Scheme 1) with the ability of active targeting for leukemia therapy was designed and fabricated based on 6-MP and the folate receptor on the surface of leukemia cells. PTA was firstly synthesized by Michael addition reaction of 6-MP with propionic acid, and then conjugated to CMCS with ethanediamine as a connecting arm to form an amphiphilic polymer prodrug. The as-prepared prodrug self-assembled into nanoparticles via ultrasonic treatment in DI water, and then was modified by folate to obtain folate receptor-targeted and GSH-responsive nanoparticles. Folate, immobilized on the surface of nanoparticles, was responsible for increasing the cell internalization and targeting the specific cancer cells and tumours. Resulting conjugates were analyzed by Fourier transform infrared spectroscopy (FTIR) and UV-visible spectrophotometer (UV-vis). The structure and morphology of the nanoparticles were identified using a transmission electron microscope (TEM) and dynamic light scattering (DLS). A drug release study was performed in media containing different GSH concentrations at pH 7.4 and pH 5.0, in vitro. Finally, cytotoxicity as well as the cellular uptake of nanoparticles against HL-60 cells and healthy cells were assessed in vitro.

## 2. Experimental

### 2.1. Materials

CMCS (M_W_ was 6.0 × 10^5^ Da defined by intrinsic viscosity measurement, degree of substitution of the carboxymethyl group and deacetylation were approximately 85% and 90%, respectively) was purchased from Yuhuan Meilunn Biochemical Co. Ltd. (Dalian, China). 1-Hydroxy-2,5-pyrrolidinedione (NHS), 3-ethylcarbodiimide hydrochloride (EDC·HCl), Glutathione (GSH) was bought from Energy Chemistry Co. Ltd. (Shanghai, China). 6-Mercaptopurine was purchased from Bide Pharmatech Ltd. (Shanghai, China). HL-60 and L929 cells were obtained from the China Center for Type Culture Collection (Wuhan University, Wuhan, China). Roswell Park Memorial Institute 1640 (RPMI-1640), penicillin-streptomycin mixtures (100 UI/mL, respectively) and trypsin were obtained from HyClone, Thermo Fisher Scientific. Foetal bovine serum (FBS), cell counting kit-8 (CCK-8), 4,6-diamidino-2-phenylindole (DAPI) and formaldehyde were purchased from Beyotime Institute of Biotechnology (Shanghai, China). All other reagents were used as received.

### 2.2. Methods

#### 2.2.1. Preparation of Prodrug PTA

PTA synthesis was carried out using the method of Turbanova et al. [36]. 6-mercaptopurine (816 mg, 4.8 mmoL) was dispersed in 32 mL methanol, and then sodium methoxide (952 mg, 17.6 mmoL) was added under constant stirring conditions. Propionic acid (336 mg, 4.8 mmoL) was added After complete dissolution of the solid powder, the mixture was refluxed for 16 h under continuous stirring to let the elements react. The addition of 4 mL deionized (DI) water terminated the reaction. Recovering the raw product took place by HCl (1 moL/L) addition to the mixture until the complete precipitation of the white precipitates. The product was purified by successive washing with DI water. To eliminate the residual moisture, the purified product was placed in a vacuum drying oven. Finally, the product was collected, and placed in a dark 4 °C refrigerator.

#### 2.2.2. Preparation of PTA-NH_2_

PTA-NH_2_ was synthesized via amide reaction between the carboxyl group of PTAs and the amino-group of ethylenediamine in the presence of EDC·HCl and NHS. Dissolution of freeze-dried PTA (264 mg, 1.0 mmoL) in DMSO (15 mL) was followed by EDC·HCl (382 mg, 2.0 mmoL) and NHS (115 mg, 1.0 mmoL) addition to the solution. After 120 min activation, an excess of ethylenediamine (72 mg, 1.2 mmoL) was added. The reaction was stirred at room temperature for 2 days, and the crude product was obtained by precipitating with dichloromethane. Then, to get the pure product, the crude product was washed several times with DI water and dried in a drying box.

#### 2.2.3. Synthesis of CMCS-g-PTA

PTA-NH_2_ was conjugated to carboxyl groups of CMCS via amide reaction. CMCS (221 mg, 1.0 mmoL) was dissolved in 5 mL DI water. EDC·HCl (382 mg, 2.0 mmoL) and NHS (115 mg, 1.0 mmoL) were added under stirring to activate the carboxyl groups. After 2 h, PTA-NH_2_ (264 mg, 1.0 mmoL) was dissolved in 8 mL dimethyl sulfoxide (DMSO), and then added dropwise into the mixture to react under stirring for 2 days at room temperature. The obtained mixture was dialyzed for 48 h with a mixed solvent of ionized water and DMF (MWCO 3500), and then DI water to eliminate the by-products and excess reactants. Obtaining the flocculation product was achieved by dialysate lyophilisation. According to their mole ratio, the collected products were named as CMCS-g-PTA-1, CMCS-g-PTA-2, and CMCS-g-PTA-3.

#### 2.2.4. Characterization of CMCS, PTA-NH_2_, CMCS-g-PTA

A Varian 500 spectrometer (AVANCE III 500MHz, Varian, Palo Alto, CA, USA) and Fourier transform infrared spectroscope (Avator360, Nicolet, MA, USA) were applied to obtain ^1^H-NMR and FT-IR spectra and facilitate the analysis of the synthesized polymer chemical structure.

#### 2.2.5. Preparation and Characterization of Surface Folate-Modified Nanoparticles

CMCS-g-PTA nanoparticles were prepared by the ultrasonic dispersion method. The CMCS-g-PTA sample was inserted in buffer (PBS, pH 7.4) and then placed in a thermostatic tank and oscillated overnight at 37 °C, 50 r/min and then sonicated for 3 min at 100 W using a probe-type sonifier (JY92-2D, Ningbo Scientz Biotechnology Co., Ltd., Ningbo, China). The dispersed solution was passed through 0.45 um filtration membrane to remove the impurities. The solution of CMCS-g-PTA nanoparticles was obtained.

Surface folate-modified nanoparticles were prepared as follows. Briefly, folate (50 mg) was dissolved in DMSO (20 mL). The carboxylic group of folates was activated by EDC and NHS addition with a FA: EDC: NHS molar ratio of 2:2:1, followed by stirring at 30 °C in the dark. After 2 h activation, the CMCS-g-PTA nanoparticles (20 mg) were added, followed by stirring the reaction system at 25 °C for 48 h. The solution was purified by dialysis with DI water to remove the impure substances. After dialysis (MWCO 3500), the unreacted folate was removed, and then the folate content in dialysis water was determined by ultraviolet spectrophotometry. The folate content of the sample was obtained by calculation. The results presented the mass content of 18% for folate and confirmed the nanoparticles’ stability.

#### 2.2.6. Characterization of Nanoparticles

A Varian 500 spectrometer (Varian, Palo Alto, CA, USA) and Fourier transform infrared spectroscope (Avator360, Nicolet, MA, USA) were applied to obtain ^1^H-NMR and FT-IR spectra for the analysis of the surface chemical structure of the modified nanoparticles. The average size, PDI and zeta potential of nanoparticles were characterized by DLS instrument (PSS NICOMP Particle Sizing System, Santa Barbara, CA, USA).

#### 2.2.7. Stability Studies of Nanoparticles

Nanoparticles stability was investigated by DLS. Briefly, 5 mg of nanoparticle powder was dissolved in pH7.4 or 6.5 PBS buffer with 20 μM GSH to form a 1 mg/mL nanoparticle solution, and then placed in an incubator (60 rpm, 37 °C) for 10 days. The particle size and size distribution were measured every day.

#### 2.2.8. Determination of Drug Content

FA-CMCS-g-PTA nanoparticles were dissolved in DI water. The content of the conjugated 6-MP was determined by ultraviolet spectrophotometry (UV 1101, Shanghai Techcomp Instrument, Shanghai, China) at 256 nm. The drug loading capacity (DLC) of the FA-CMCS-g-PTA nanoparticles was obtained using the following equation:

$$\mathrm{DLC}\left(\%\right)=\left(\frac{C_{6-MP}\times V_{solution}}{m}\right)\times 100$$

where *C*_6-*MP*_ is the concentration of the conjugated 6-MP in nanoparticle solution according to the standard curve, *V_solution_* is the volume of the nanoparticle solution, and m is the weight of FA-CMCS-g-PTA nanoparticles.

#### 2.2.9. In Vitro Drug Release Study

The 6-MP release from nanoparticles was studied using the dialysis method in PBS (pH 5.0, 6.5 and 7.4). 5 mg of the FA-CMCS-g-PTA NPs was dissolved in 5 mL of the PBS solutions containing different GSH concentrations (20 μM, 2 mM, 10 mM, 20 mM). The solutions were transferred to a dialysis bag (molecular weight cut off: 3500) followed by immersion in 45 mL of corresponding medium and oscillation for 48 h in a shaking bed at 100 rpm at 37 °C. At different intervals, 2 mL of the release medium was withdrawn and characterized by UV. Using the following equation and the calibration curve, the 6-MP cumulative release was obtained:

$$\mathrm{Cumulative}\;\mathrm{release}\;\left(\%\right)=\frac{2\times \sum_{i-1}^{n-1}C_{i}+45\times C_{n}}{\mathrm{weight}\;\mathrm{of}\;6-\mathrm{MP}\;\mathrm{in}\;\mathrm{the}\;\mathrm{nanoparticles}}\times 100$$

which *C_i_* and *C_n_* represent the mass concentration of 6-MP at time *i* and *n*, respectively.

#### 2.2.10. In Vitro Cytotoxicity Studies

In vitro cytotoxicities were assessed by using the Cell Counting Kit-8 (CCK-8) assay. Referring to document [16], HL-60 cells and L929 cells were selected for cytotoxicity studies in vitro. HL-60 cells and L929 were cultured in 10% fetal bovine serum (FBS), 100 units/mL penicillin and 100 μg/mL streptomycillin added minimum essential medium (MEM) with 5% CO_2_ and 95% relative humidity at 37 °C. The cells were collected, seeded in a 96-well tissue culture plate at a density of 5 × 103 cells/well and allowed to adhere overnight. Serial dilutions of the filtered samples (FA-CMCS-g-PTA and CMCS-g-PTA) with varying concentrations (0.1–50 μg 6-MP equiv/mL) or medium alone were added to test cells and afterwards, the cells were cultured for 24 h and 48 h, respectively. After the addition of CCK-8 (10 μL) to each well, and a second cell incubation for 2 h, the optical density evaluation for the solution was performed at 550 nm. The relative cell viability in comparison with the control wells (cells only) was also determined.

#### 2.2.11. Cellular Uptake of Nanoparticles

The cellular uptake of CMCS-g-PTA and FA-CMCS-g-PTA NPs by HL-60 cells was estimated by CCK-8 assay. Since the raw material is unable to spontaneously display fluorescence (red), it was marked here with Nile red. HL-60 cells (5 × 10^4^ cells/well) were incubated in 12-well cell culture plate overnight. The next day, cells were treated with two different nanoparticles containing equivalent amounts of 6-MP for 0, 1, 2, and 4 h. After removing the medium and, washing the cells with PBS (pH 7.4), 4% *W*/*V* formaldehyde was added to immobilize the cells for 20 min and the cells underwent another washing step using PBS. Thereafter, cells were stained with DAPI for 15 min. At the specific time points, the cellular uptake was studied by confocal microscopy after washing the cells by PBS. Nile red was excited at 514 nm with emission at 630 nm.

#### 2.2.12. Flow Cytometry Analysis

HL-60 cells and L929 cells were inoculated into 6-well plates. Untreated cells served as controls. Centrifugation helped to remove the culture medium and the fresh medium containing CMCS-g-PTA or FA-CMCS-g-PTA NPs was added at the same dose of 6-MP (2 μg/mL). After incubation for 1 or 4 h, the cells were washed with PBS and centrifuged (1000 rpm) for 5 min. Then, the cells were collected and re-suspended in PBS (1 mL). Finally, the cells were suspended in a centrifuge tube after washing twice and further analysed by flow cytometry (Becton, Dickinson and Company, Franklin Lakes, NJ, USA).

## 3. Results and Discussion

### 3.1. Synthesis and Characterization of CMCS, PTA, PTA-NH_2_, and CMCS-g-PTA

**Scheme 2:**
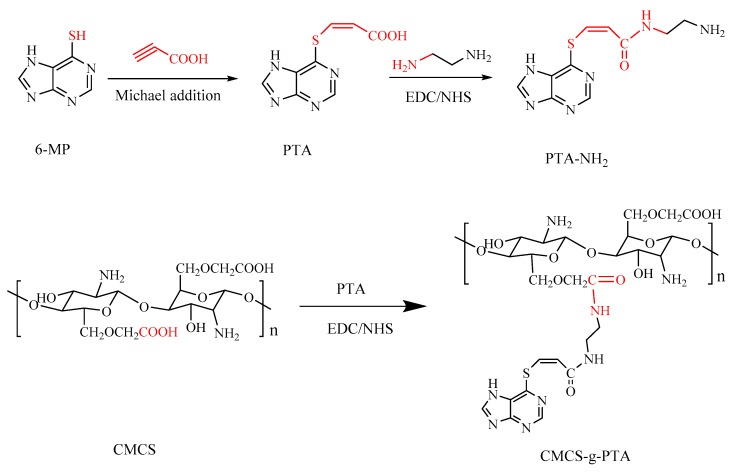
Schematic illustration of the synthesis route of CMCS-g-PTA conjugate.

CMCS-g-PTA was synthesized as illustrated in Scheme 2. First, PTA was synthesized via Michael addition with 6-MP and propiolic acid. Then PTA-NH_2_ was formed via the amide reaction between the carboxyl group of PTA and the amino-group of ethylenediamine. Finally, the obtained PTA-NH_2_ was conjugated to CMCS via an amidation reaction to obtain CMCS-g-PTA prodrug. Characterization of the prodrug and some intermediate products was achieved using ^1^H-NMR and FT-IR spectroscopy.

**Figure 1:**
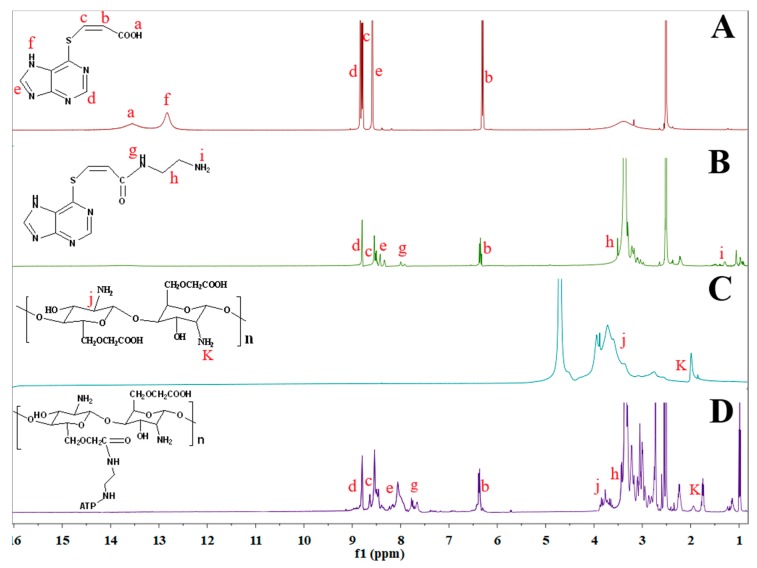
^1^H NMR spectra of PTA (**A**); PTA-NH_2_ (**B**); CMCS (**C**); CMCS-g-PTA (**D**).

The chemical structures of PTA, PTA-NH_2_, CMCS and CMCS-g-PTA were characterized by ^1^H NMR. As shown in Figure 1, all the labelled proton assignments correspond well to the molecular structure. The proton signals of PTA are assigned as follows (ppm): 13.55 (s, 1H), 12.80 (s, 1H), 8.83 (s, 1H), 8.58(s, 1H), 8.79 (d, J = 10.1 Hz, 1H), 6.31 (d, J = 10.1 Hz, 1H), and the peaks located at δ = 6.31, 8.79 and 13.75 ppm were ascribed to the chemical shifts of C=C and COOH, which confirms the formation of PTA. Compared to curve A, the characteristic absorption peak of ethylenediamine was observed in curve B and the signal peaks of carboxyl at 13.55 ppm disappeared. The signal peak at 7.75 ppm, attributed to the O=C-NH protons, is an indication of the amide reaction between the carboxyl groups in PTA and the amine group in ethylenediamine. Compared with the spectrum of CMCS (curve C), curve D presented new characteristic signals at 8.80 and 8.42 ppm (purine ring protons of MP) as well as 6.35 and 8.50 ppm(C=C), which were respectively assigned to the purine ring protons of MP and the HC=CH protons, indicating the linkage of CMCS and PTA-NH_2_.

**Figure 2:**
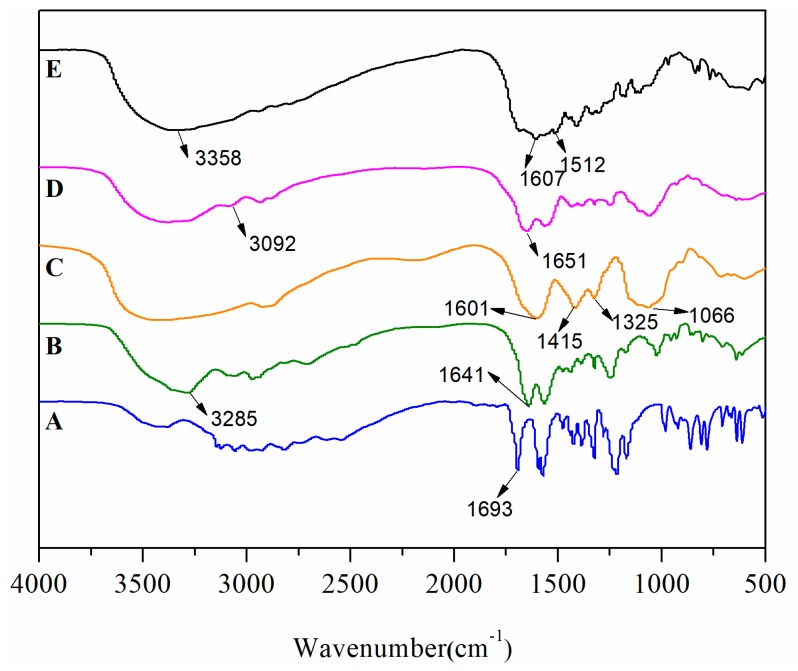
FT-IR characterization: PTA (**A**); PTA-NH_2_ (**B**); CMCS (**C**); CMCS-g-PTA (**D**); FA-CMCS-g-PTA (**E**).

FT-IR also confirmed the successful synthesis of CMCS-g-PTA. For reference [16], the bands of 6-MP were assigned as follows: 3440 cm^−1^ (N–H, stretch), 2670 cm^−1^ (S–H, stretch), 1610 cm^−1^ (amide I band), 1600–1200 cm^−1^ (purine ring). Compared to 6-MP, a new peak appears at 1693 cm^−1^ (–C=O, stretch) and the intensity at 2670 cm^−1^ decreases in curve A, confirming the link formed between 6-MP and propiolic acid, thereby PTA was successfully synthesized. Compared to the PTA spectrum, there is no characteristic absorption peak of the carboxyl group at 1693 cm^−1^ on curve B, but a characteristic peak of the amido bond appears at 1641 cm^−1^, which is a confirmation of the successful PTA-NH_2_ synthesis. Compared to CMCS (curve C in Figure 2), in curve D some new obvious peaks appeared in 1200–1600 cm^−1^ as well as the presence of a peak at 3092 cm^−1^, demonstrating that CMCS-g-PTA was successfully synthesized.

### 3.2. Preparation and Characterization of Surface Folate-Modified Nanoparticles

The amphiphilic structure of CMCS-g-PTA prodrug due to the hydrophobic PTA-NH_2_ moieties and hydrophilic CMCS backbone, facilitates self-assembly of the prodrug into the nanoparticles. The free amine groups of the polymer present on the surface of the obtained nanoparticles (CMCS-g-PTA NPs) can conjugate to the COOH-containing compounds to form the surface functionalized nanoparticles. Moreover, the surface of CMCS-g-PTA NPs was modified by folate to obtain FA-CMCS-g-PTA NPs with targeting capability.

**Figure 3:**
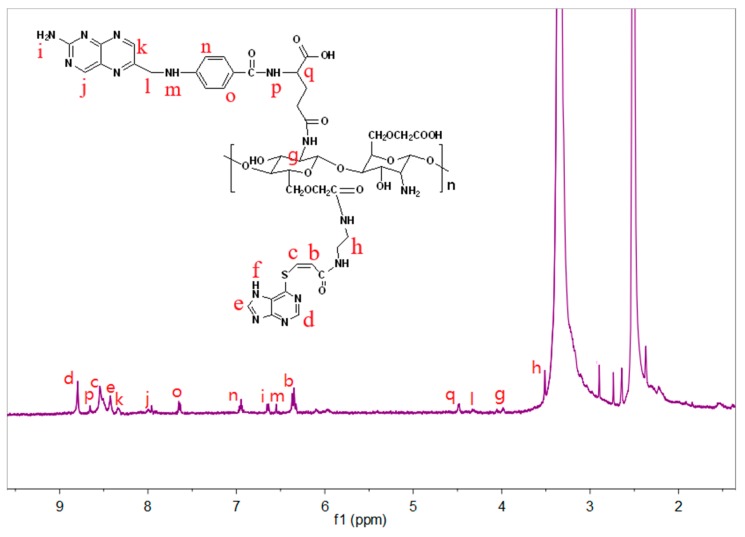
^1^H NMR spectra of FA-CMCS-g-PTA NPs.

Nuclear magnetic resonance and infrared spectroscopy were utilized to characterize the conjugation of folate to the surface amine groups of CMCS-g-PTA NPs. As shown in Figure 3, the signals corresponding to protons of the structure of FA-CMCS-g-PTA have been marked with the same alphabet. The signals at 7.66 and 6.95 ppm ascribed to benzene ring protons of FA and signals at 8.34 and 8.66 ppm is assignable to pteridine protons of FA. It is indicated that FA was successfully conjugated to the surface of CMCS-g-PTA nanoparticles. In addition, the FT-IR spectra clearly affirmed the successful surface modification of nanoparticles. As depicted in Figure 2E, the characteristic absorption peaks of FA at 1569 cm^−1^ (benzene skeleton vibration), 1684 cm^−1^ (OH–C=O, stretch), and 3358 cm^−1^ (–NH, stretch) were observed compared to IR-spectrum of CMCS-g-PTA.

**Figure 4:**
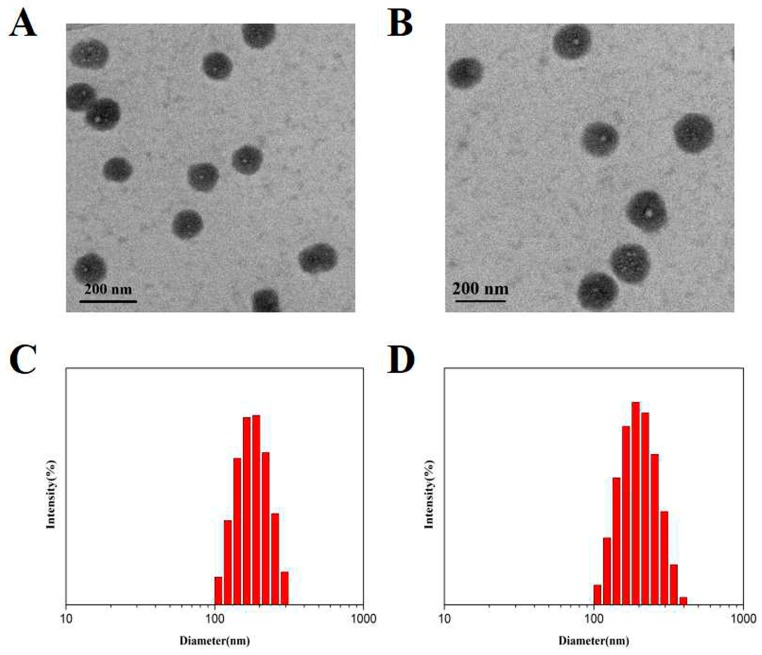
TEM images of CMCS-g-PTA-3 NPs (**A**) andFA-CMCS-g-PTA-3 NPs (**B**). DLS histograms of CMCS-g-PTA-3 NPs (**C**) and FA-CMCS-g-PTA-3 NPs (**D**).

The morphologies and size distribution of CMCS-g-PTA NPs and FA-CMCS-g-PTA NPs were observed by TEM and DLS. As shown in Figure 4A,B, using TEM, these nanoparticles are spherical with a relatively uniform size of about 132 nm (Figure 4A) and 186 nm (Figure 4B) for CMCS-g-PTA NPs and FA-CMCS-g-PTA NPs, respectively. The slightly smaller sizes determined by TEM compared to that of DLS (189 nm and 212 nm for CMCS-g-PTA NPs and FA-CMCS-g-PTA NPs, respectively), suggesting that the nanoparticles can swell in aqueous media.

To evaluate the effect of drug content on particle size, a series of nanoparticles containing different drugs were synthesized. The measurement of their mean particle size, PDI and zeta potential were performed using DLS. As shown in Table 1, the size of surface folate-modified samples (FA-CMCS-g-PTA NPs) ranges from 183 to 216 nm, while the size of no folate modified samples (CMCS-g-PTA NPs) varies in the range of 173–199 nm, showing that the folate conjugation did not significantly alter the nanoparticle size. Moreover, drug content increased results in slight enhancement of FA-CMCS-g-PTA NPs and CMCS-g-PTA NPs diameters, indicating that the drug content and the particle size have direct correlation. In addition, all samples had the zeta potential in the range of −13.0 m to −8.0 mV and showed a slight difference (see Table 1), demonstrating that the drug content and folate modification had no significant effect on electric potential.

**Figure 5:**
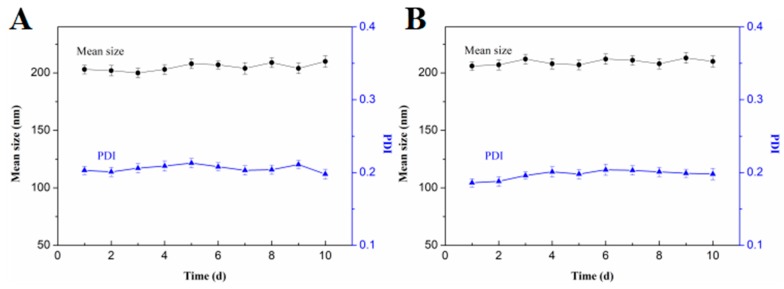
Changes of mean size and PDI of FA-CMCS-g-PTA-3 NPs for 10 days, the nanoparticles incubation in the PBS 7.4 (**A**) and 6.5 (**B**) solution with 20 μM GSH.

To examine the colloidal stability of FA-CMCS-g-PTA NPs and CMCS-g-PTA NPs, the alteration of their particle sizes and distributions with time were also obtained by DLS [37]. Figure 5 shows that neither the average particle size nor the size distribution change significantly in PBS at pH 7.4 and 6.5 with 20 μM of GSH for 10 days. This indicates that the nanoparticles were stable, and this feature improves the resistance of the nanoparticles against 6-MP leakage during storage and blood circulation.

### 3.3. In Vitro Drug Release

**Figure 6:**
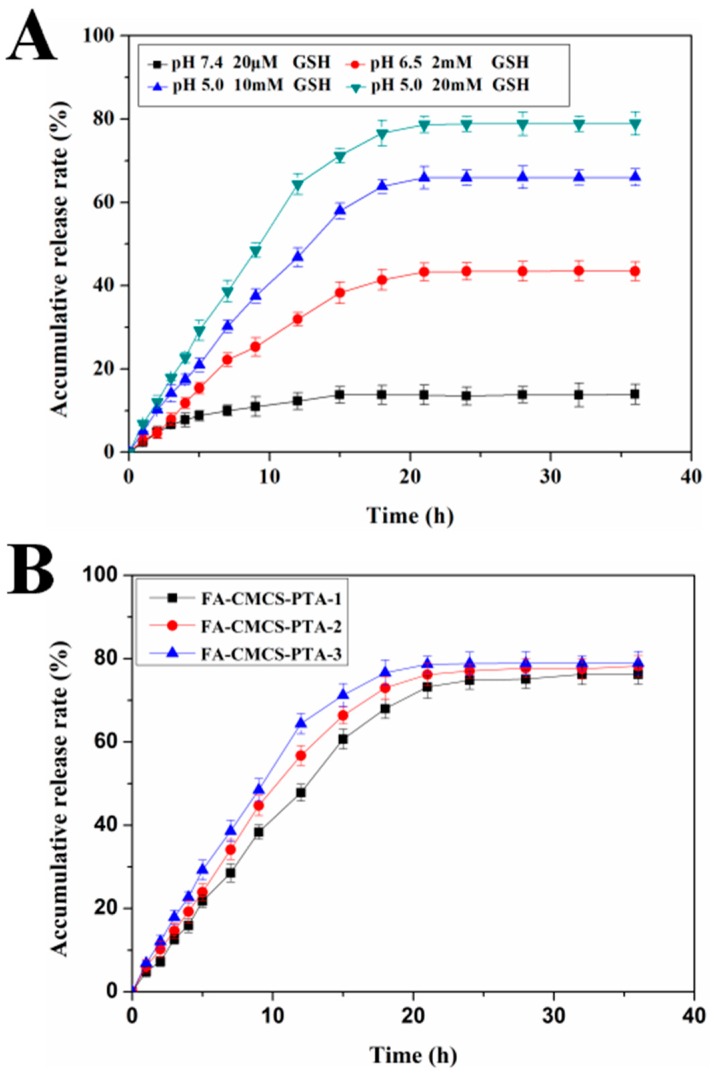
(**A**) Release profiles of 6-MP from FA-CMCS-g-PTA-3 NPs in different concentrations of GSH media over 36 h. The results represent the mean ± SD (n = 3). (**B**) The release of 6-MP from FA-CMCS-g-PTA NPs with different drug content in pH 5.0 buffer containing 20 mM GSH.

Rapid drug release in the target cells and avoiding random release in the circulatory system and normal cells is of a vital importance for designing an ideal drug delivery system. Four media were selected for drug release tests: PBS at pH 7.4 with the addition of 20 μM GSH (simulating the environment in human blood); PBS at pH 6.5 with the addition of 2 mM GSH (intracellular milieu of normal cells) and PBS at pH 5.0 with the addition of 10 mM or 20 mM GSH (intracellular milieu of cancer cells). The 6-MP release from FA-CMCS-g-PTA NPs in the four media is shown in Figure 6A. After 36 h incubation of nanoparticles at pH 7.4 with 20 μM GSH, a drug release rate of less than 14% was observed, showing that nanoparticles are chemically stable. Furthermore, even after increasing the GSH concentration to 2 mM at pH 6.5, the drug release rate was still slow, and the cumulative release was only 39.5% in 18 h. Decreasing the pH of the buffer to 5.0 and increasing the GSH concentration to 10 mM and 20 mM resulted in a rapid release of 6-MP from the nanoparticles and higher cumulative release rate of about 65.9% and 78.8% at 18 h, respectively. This phenomenon indicates that GSH stimulation could enhance the release of the drug and increase the selective release in tumour cells.

The 6-MP release depends on a Michael addition-elimination reaction. More specifically, electron transfer occurs due to the GSH attacks to the β-C of the unsaturated bond. GSH also chemically combines with the polymeric prodrug and by forming a link between H+ and 6-MP residue 6-MP is released. Thus, GSH concentration plays a significant role in the release of 6-MP from the nanoparticles. The high concentration of GSH results in more exchange of 6-MP and its fast release.

The drug content also influences the drug release. Figure 6B depicts the release profiles of surface modified nanoparticles with distinct 6-MP contents in 20 mM GSH added PBS at pH 5.0.

In the first 20 h, it is obvious that there is a certain difference in the cumulative release rate of 6-MP for the nanoparticles containing different drug content. It is indicated that the content of 6-MP in nanoparticles affects the release rate of drugs. The possible reason is that by increasing the drug content, the nucleus in the hydrophobic region of nanoparticles increases, which makes it easier for more glutathione to contact with the α, β unsaturated bonds, thus, increasing the drug release rate.

### 3.4. In Vitro Cytotoxicity

**Figure 7:**
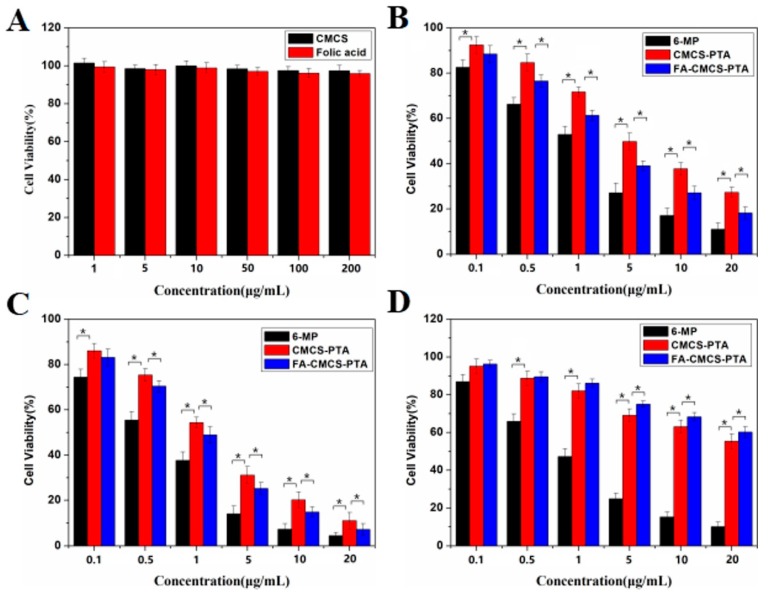
Cytotoxicity of CMCS and FA against L929 cells (**A**) Cytotoxicity of 6-MP, CMCS-g-PTA NPs and FA-CMCS-g-PTA NPs against L929 cells after 48 h incubation (**B**), and against HL-60 cells after 24 h (**C**), and 48 h (**D**) incubations. The error bars represent means SD (n = 6). * *p* ≤ 0.001.

The cytotoxicity evaluation was performed using CCK-8 methods. L929 cells were used as controls, and HL-60 cell lines were utilized to evaluate the anti-leukemia effect in vitro. Media containing L929 and HL-60 cells were used to incubate all the samples for 48 h before cell viability rate measurement. As shown in Figure 7A, low cytotoxicity against L929 cell lines was observed for blank CMCS and folate (1 to 200 μg mL^−1^) after 48 h of incubation. Even after increasing the concentration of CMCS and folate solution to the highest level of 200 μg/mL, the cell viability rate remained above 95%. So, CMCS and folate have acceptable biocompatibility for L929 and HL-60 cells.

As displayed in Figure 7C, the inhibition rate of FA-CMCS-g-PTA NPs on HL-60 cells increases significantly in the concentration range of 0.1–20 mg/L. At the highest concentration, the inhibition rate of FA-CMCS-g-PTA NPs is close to the free 6-MP. In addition, compared to the unmodified CMCS-g-PTA NPs, FA-CMCS-g-PTA NPs showed a lower cell viability rate. The IC_50_ values for CMCS-g-PTA NPs incubated with HL-60 cells were 4.75 and 1.57 μg/mL at 24 and 48 h, respectively. Nevertheless, the IC_50_ values for FA-CMCS-g-PTA NPs were 2.34 and 0.80 μg/mL, at the corresponding times, respectively. This may be attributed to the 6-MP release triggered by the high GSH concentration in cancer cells and the high affinity of folate-modified nanoparticles to tumour cells. Folate-modified nanoparticles can be recognized by β-folate receptors on the tumour cell surface, and thus, enter tumour cells via the receptor mediated mechanism and release 6-MP in response to a high concentration of GSH, leading to a higher cell death rate.

In contrast to free 6-MP, the survival rate of L929 cells treated with FA-CMCS-g-PTA NPs for 48 h was significantly higher (see Figure 7D), suggesting that the modified nanoparticles have lower toxicity to the normal cells. Interestingly, FA-CMCS-g-PTA NPs showed a lower cytotoxicity than CMCS-g-PTA NPs against the L929 cells. The possible reason is that the surface modification of CMCS-g-PTA NPs increases the particle size and makes it difficult to enter L929 cells. This result revealed that the FA-CMCS-g-PTA NPs may have low side effects on normal cells.

### 3.5. Cellular Internalization of Nanoparticles In Vitro

**Figure 8:**
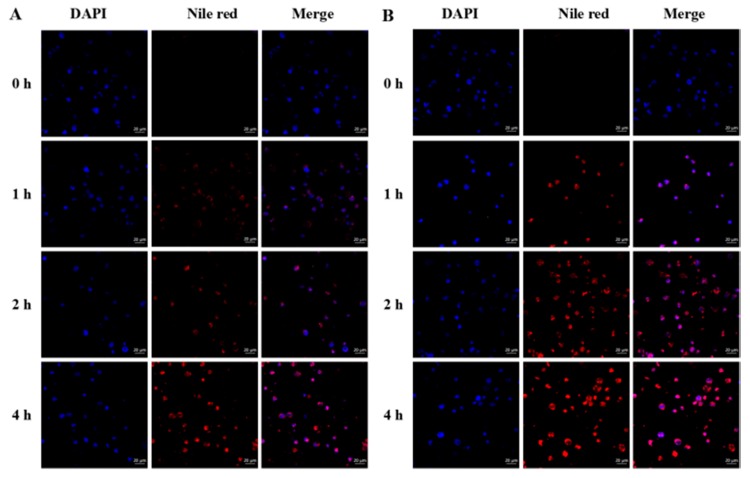
Confocal laser scanning microscopy-images of HL-60 cells incubated with CMCS-g-PTA NPs (**A**) and FA-CMCS-g-PTA NPs (**B**) for 0, 1, 2 and 4 h.

The specific binding between folate and folate receptor on the cell surface could enhance the cytotoxicity of nanoparticles against tumour cells. Therefore, the cellular uptake of the surface-modified FA-CMCS-g-PTA nanoparticles was investigated by CLSM. Nile red (NR), a hydrophobic fluorescent molecule, was encapsulated inside the nanoparticles to track the cellular nanoparticle uptake. Nanoparticles enter cancer cells and respond to high concentrations of GSH, which promotes drug release from nanoparticles. Disintegration of the hydrophobic core inside the nanoparticles is followed by the release of NR. Therefore, red fluorescence is detectable. Figure 8 represents CLSM images of the 4 h incubation of HL-60 cells with NR-loaded FA-CMCS-g-PTA nanoparticles. The blue fluorescence represents the cell nuclei position using DAPI as the stain. By increasing the culture time, strong red fluorescence was detectable around the nucleus, demonstrating that the nanoparticles were effectively uptaken by cells and confirming the effectiveness of the nanoparticles for HL-60 cell delivery.

To demonstrate the targeting ability of nanoparticles, the non-targeted nanoparticles (CMCS-g-PTA NPs) were also incubated with HL-60 for 4 h. It is clearly observed in Figure 8 that the red fluorescence intensity of the CMCS-g-PTA NPs in the HL-60 is weaker than the FA-CMCS-g-PTA NPs at every time point. After incubation for 1 h, the red fluorescence of the FA-CMCS-g-PTA NPs was observed on the nuclear surface, while the fluorescence of the CMCS-g-PTA NPs appeared after 2 h. This indicated that the folate-targeted nanoparticles could significantly promote their internalization into the HL-60.

**Figure 9:**
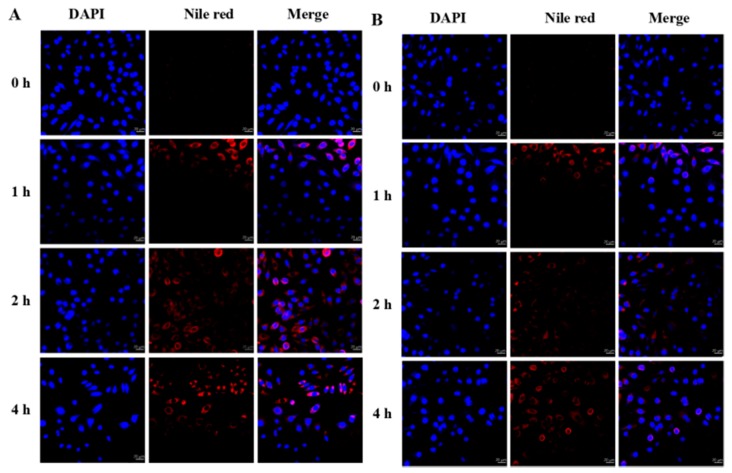
Confocal fluorescence microscopy pictures of CMCS-g-PTA NPs (**A**) and FA-CMCS-g-PTA NPs (**B**) taken up by L929 cells for 0, 1,2 and 4 h.

Besides, the cellular uptake of nanoparticles into normal cells was also examined as a comparison (Figure 9). L929 cells were incubated with FA-CMCS-g-PTA nanoparticles for 4 h. As shown in Figure 9, a slightly weaker intracellular fluorescence was observed compared to the HL-60 tumour cells incubated with unmodified CMCS-g-PTA NPs. However, compared to the HL-60 tumour cells incubated with FA-CMCS-g-PTA NPs, the fluorescence was significantly weaker. This indicated that both the level of folate receptor on the cell surface and the intracellular GSH concentration affected the drug release rate. Because of the undetectable level of folate receptor at the normal cell surface and the lower intracellular GSH, the intracellular uptake of nanoparticles decreased, and the intracellular drug release also decreased, which results in significantly weaker fluorescence. These results further confirm the potential of FA-CMCS-g-PTA NPs for tumour targeted drug delivery.

### 3.6. Flow Cytometric Profiles

**Figure 10:**
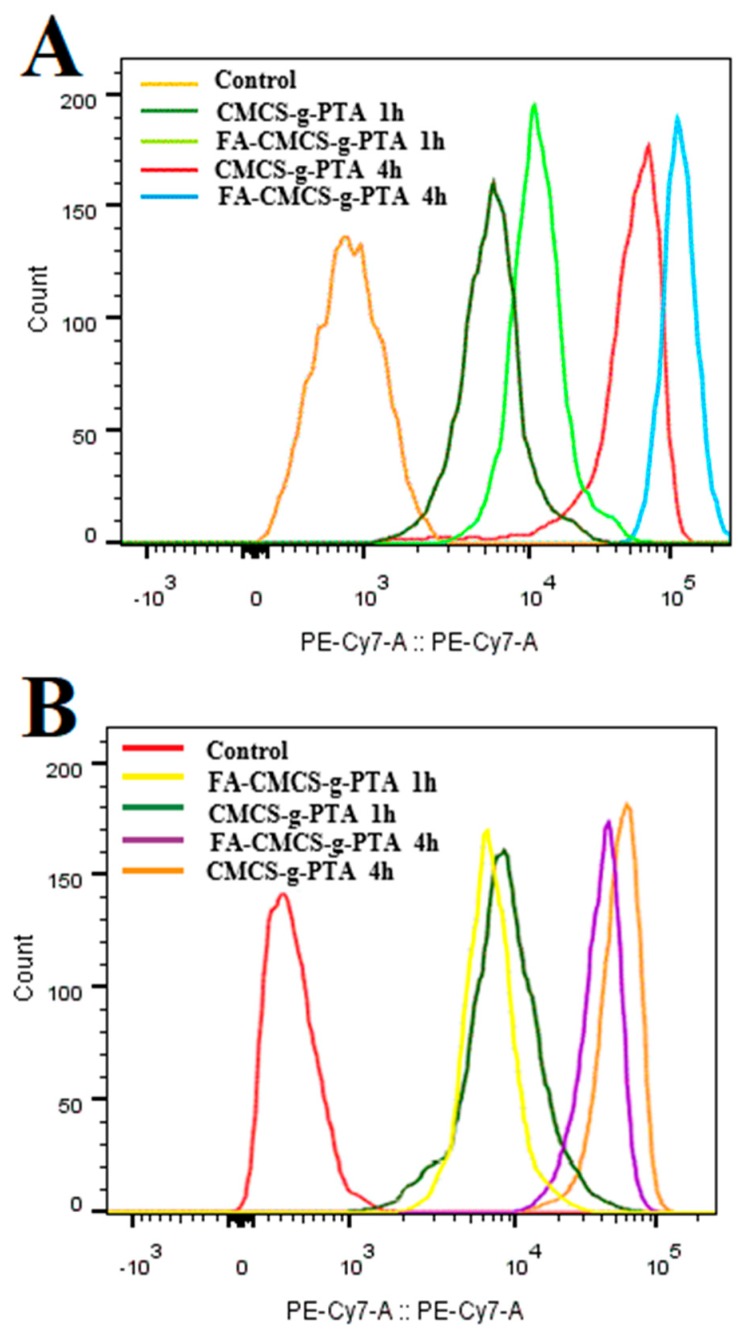
Flow cytometric profiles of HL-60 cells (**A**) and L929 (**B**) incubated with CMCS-g-PTA NPs and FA-CMCS-g-PTA NPs for 1 or 4 h.

The fluorescence intensity of Nile red in HL-60 cells and L929 cells after 1 and 4 h incubation was evaluated by flow cytometry. HL-60 cells were supplemented with two specific nanoparticles (CMCS-g-PTA NPs and FA-CMCS-g-PTA NPs) and the cells without any treatment were used as control. As displayed in Figure 10, the fluorescence intensity of the HL-60 cells treated with FA-CMCS-g-PTA NPs was higher than that of the HL-60 cells treated with CMCS-g-PTA NPs. However, for normal L929 cells, the relative geometric fluorescence intensity after being treated with FA-CMCS-g-PTA NPs was significantly weaker than that after being treated with CMCS-g-PTA NPs. These results further confirmed that FA-CMCS-g-PTA NPs can target tumour cells, enhance drug release and aggregation in target cells, and reduce the side effects on normal cells, and thus, FA-CMCS-g-PTA NPs has desirable application prospects in tumour therapy.

## 4. Conclusions

In conclusion, a folate-targeted nano-drug delivery system with GSH-dependent drug release was successfully developed. CMCS-g-PTA prodrugs were synthesized by attaching 6-MP to the carboxyl group of carboxymethyl chitosan via a GSH-sensitive carbonyl vinyl sulfide linkage. The prodrugs can self-assemble into GSH-responsive nanoparticles owing to their amphiphilic structure in aqueous solution. By folate surface modification, the CMCS-g-PTA nanoparticles were endowed with folate receptor targeting. An important advantage of this system for cancer treatment is the small amount of drug leakage in the simulated circulatory system and the large quantity of release in the tumour cell environment. It is proved that the cumulative release of drugs is regulated by GSH concentration. More importantly, selective internalization of the nanoparticles by cancer cells with high over-expression of folate receptor is demonstrated. The nanoparticle system could highly inhibit the HL-60 cell proliferation and showed a considerably lower cytotoxicity for normal L929 cells. Therefore, folate receptor targeting and GSH-responsive carboxymethyl chitosan nanoparticles have good potential application prospects. On this basis, we plan to carry out animal experiments to evaluate the anti-tumor effect in vivo.

## Figures and Tables

**Table 1:** Characterization of the nanoparticle’s formulas formed by different weights of CMCS.

Samples	Feed Ratio ^a^ (mole)	Size (nm)	PDI	Zeta Potential (mV)	Grafting Ratio of PTA (%)	DLC (6-MP, *w*/*w* %)
CMCS-g-PTA-1	1:1.0	182 ± 4	0.214	−9.47 ± 0.22	11.7	7.1
CMCS-g-PTA-2	1:2.0	191 ± 3	0.208	−11.7 ± 0.36	14.6	8.5
CMCS-g-PTA-3	1:4.0	203 ± 7	0.223	−13.6 ± 0.30	17.9	10.1
FA-CMCS-g-PTA-1	1:1.0	191 ± 5	0.257	−8.36 ± 0.37	11.7	6.7
FA-CMCS-g-PTA-2	1:2.0	201 ± 4	0.245	−10.7 ± 0.26	14.6	7.8
FA-CMCS-g-PTA-3	1:4.0	210 ± 6	0.263	−12.9 ± 0.34	17.9	9.0

Notes: ^a^ The molar ratio between alkynyl groups of CMCS and azido groups of PTA.
